# Applications of the Critical Power Model to Dynamic Constant External Resistance Exercise: A Brief Review of the Critical Load Test

**DOI:** 10.3390/sports9020015

**Published:** 2021-01-21

**Authors:** Haley C. Bergstrom, Taylor K. Dinyer, Pasquale J. Succi, Caleb C. Voskuil, Terry J. Housh

**Affiliations:** 1 Department of Kinesiology and Health Promotion, University of Kentucky, Lexington, KY 40506, USA; taylor.dinyer@uky.edu (T.K.D.); pj.succi@uky.edu (P.J.S.); caleb.voskuil@uky.edu (C.C.V.); 2 Department of Nutrition and Health Sciences, University of Nebraska-Lincoln, Lincoln, NE 68583, USA; thoush1@unl.edu

**Keywords:** critical power, critical load, dynamic constant external resistance exercise, fatigue, exercise intensity domains

## Abstract

The study and application of the critical power (CP) concept has spanned many decades. The CP test provides estimates of two distinct parameters, CP and W′, that describe aerobic and anaerobic metabolic capacities, respectively. Various mathematical models have been used to estimate the CP and W′ parameters across exercise modalities. Recently, the CP model has been applied to dynamic constant external resistance (DCER) exercises. The same hyperbolic relationship that has been established across various continuous, whole-body, dynamic movements has also been demonstrated for upper-, lower-, and whole-body DCER exercises. The asymptote of the load versus repetition relationship is defined as the critical load (CL) and the curvature constant is L′. The CL and L′ can be estimated from the same linear and non-linear mathematical models used to derive the CP. The aims of this review are to (1) provide an overview of the CP concept across continuous, dynamic exercise modalities; (2) describe the recent applications of the model to DCER exercise; (3) demonstrate how the mathematical modeling of DCER exercise can be applied to further our understanding of fatigue and individual performance capabilities; and (4) make initial recommendations regarding the methodology for estimating the parameters of the CL test.

## 1. Introduction

### Historical Perspective: The Influence of Dr. Herbert A. deVries

Over his 50-year career as a professor of physical education and exercise physiology, Dr. Herbert A. deVries published many landmark discoveries in areas such as the health and fitness benefits of exercise training in the elderly, the tranquilizer effect of exercise, applications of surface electromyography in fatigue and muscle function, and neural factors and hypertrophy in the time course of muscle strength gains [1,2,3,4,5]. His personal life was also full of passion for many things, including cars, motorcycles, surfing, and, perhaps most of all, aviation. Dr. deVries was an avid airplane and glider pilot. It was in his graduate course in ergonomics at the University of Southern California in the late 1970s that his passions for aviation and human performance came together and led to the development of the whole-body analogue of the critical power (CP) technique described by Monod and Scherrer [6] for continuous and intermittent static contractions of synergic muscle groups. During this time period, there was international interest in human-powered flight, driven, in part, by prize money offered by British industrialist Henry Kremer. The first Kremer Prize of $95,000 was won in 1977 by a team, led by Californian Paul MacCready, with the Gossamer Condor piloted by renowned cyclist Bryan Allen. The one-mile course took 6 min and 22 s to complete at a flight speed of 10–11 miles per hour and required a cycling power output of about 250 watts (0.33 horsepower). In 1979, MacCready and Allen teamed up to win the second Kremer Prize of approximately $190,000 for crossing the 26-mile English Channel in 2 h and 49 min in the 70-lb aircraft, the Gossamer Albatross.

The idea of human-powered flight intrigued Dr. deVries, and he began to think about how to identify the maximal rate of fatigueless work for an individual during cycle ergometry, such as that used to power the Gossamer Condor and Gossamer Albatross. Dr. deVries brought this question to the students in his ergonomics course, one of whom was Toshio Moritani, a doctoral student of Dr. deVries. An important part of Dr. Moritani’s training included completing a series of original and independent research projects, one of which was the classic 1981 paper published in the journal *Ergonomics* [7] entitled “Critical Power as a Measure of Physical Work Capacity and Anaerobic Threshold”. This study extended the work of Monod and Scherrer [6] to whole-body cycle ergometry and has served as the foundation for hundreds, perhaps thousands, of papers related to the CP concept [8,9,10,11].

The aims of this review are to (1) provide an overview of the CP concept across continuous, dynamic exercise modalities; (2) describe the recent applications of the model to dynamic constant external resistance (DCER) exercise; (3) demonstrate how the mathematical modeling of DCER exercise can be applied to further our understanding of fatigue and individual performance capabilities; and (4) make initial recommendations regarding the methodology for estimating the parameters of the critical load (CL) test.

## 2. The Modeling of Human Performance

**Figure 1:**
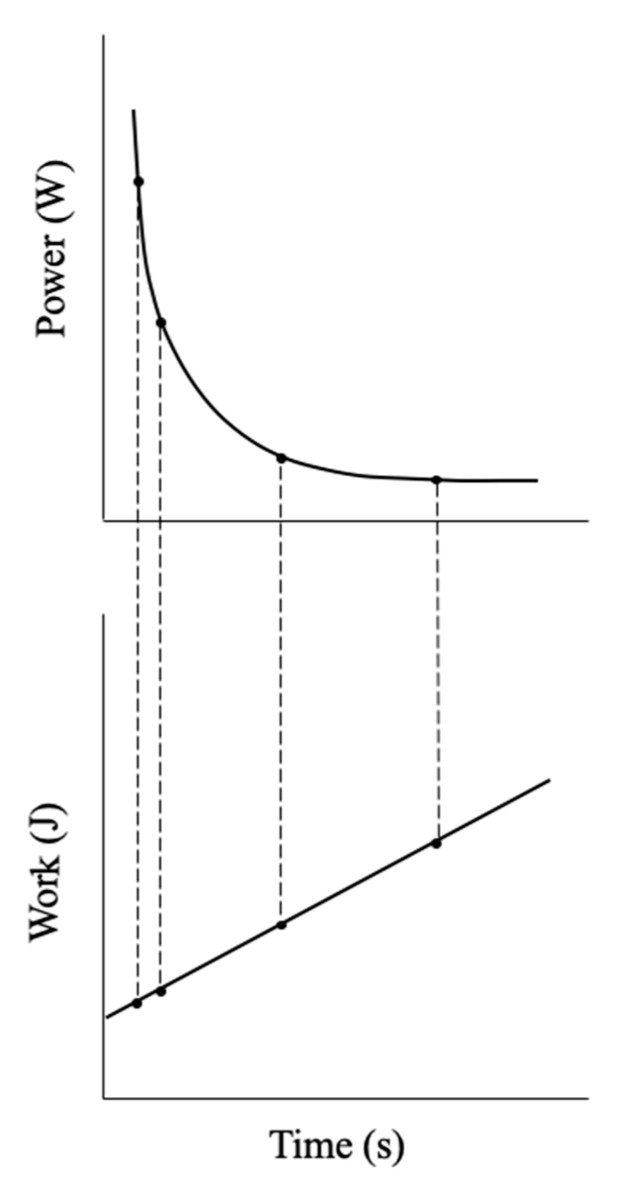
Theoretical representation of the linearization of the power output versus duration curve to derive the parameters of the critical power (CP) test. The upper panel demonstrates the negative, curvilinear relationship between power output (P) and time to exhaustion (T_Lim_) that can be used to estimate the T_Lim_ for any P that is greater than CP using the equation T_Lim_ = W′/(P − CP). The lower panel demonstrates the linear relationship between the total work completed (W_Lim_) and T_Lim_. Each work bout at a constant power output can only be maintained for a finite amount of time, which results in the completion of a finite amount of work. When the W_Lim_ and T_Lim_ are plotted against each other (lower panel), there is a linear relationship that can used to derive the parameters of the CP test. The slope is CP and the y-intercept is the W′ (W_Lim_ = CP × (T_Lim_) + W′).

The nature of continuous, dynamic whole-body exercise was first documented by A.V. Hill in 1925 from the relationship between average running, swimming, and rowing speeds (yd·s^−1^) for world records versus the time in seconds to complete the race [12]. This curvilinear, asymptotic relationship was further examined for dynamic, continuous isometric, and intermittent isometric exercise of local muscle actions (<1/3 total muscle mass), and a linear model [6] was developed from measures of the total work performed or limit work (W_Lim_) and time to exhaustion or limit time (T_Lim_) (Figure 1). Together, these parameters formed the linear equation W_Lim_ = y-intercept + slope × T_Lim_, where the slope was termed CP, which corresponded to the “maximum rate [a muscle or muscle group] can keep up for a very long time without fatigue” [6] (p. 329). The y-intercept was defined as an “energetic reserve” that is used during exercise above CP [6], which later investigators called the anaerobic work capacity (AWC) [7,8] or curvature constant (W′) [13,14,15]. Thus, Monod and Scherrer [6] expanded the early observations of A. V. Hill [12] of the curvilinear relationship between average speed and world record time and described the linear relationship between the time to exhaustion and the work performed from multiple work bouts, to define individual performance capabilities.

## 3. The Critical Power Test

**Figure 2:**
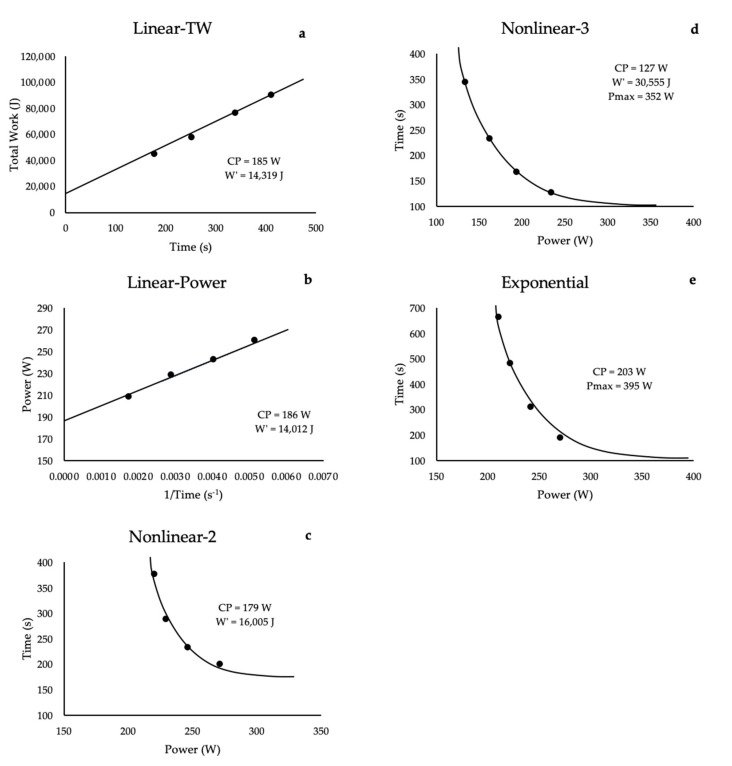
A schematic representation of the five mathematical models that have been used to estimate the parameters of the critical power (CP) test, CP and W′; (**a**) linear model based on the regression analysis of the total work (TW) versus time to exhaustion, where CP is the slope and W′ is the y-intercept; (**b**) linear model based on the regression analysis of the power output versus the inverse of time to exhaustion, where W′ is the slope and CP is the y-intercept; (**c**) nonlinear, 2-parameter regression model of time versus power output, where CP is the asymptote and W′ is the curvature constant; (**d**) nonlinear, 3-parameter regression model of time versus power output, where CP is the asymptote, W′ is the curvature constant, and maximal instantaneous power (Pmax) is the x-intercept; and (**e**) exponential regression model of time versus power output, where CP is the asymptote and Pmax is the x-intercept.

Building on the work of Monod and Scherrer [6], Moritani et al. [7] applied this CP concept to cycle ergometry, demonstrating the linear relationship between the total work performed and the time to exhaustion from various constant power output trials. A salient feature of the CP test is that it requires only a cycle ergometer and a stopwatch. As described by Monod and Scherrer [6] and Moritani et al. [7], the determination of CP typically requires three to five exhaustive, constant power output work bouts, where exhaustion is reached within 2 to 15 min [7,8,13]. The total work (W_lim_) (power output × time to exhaustion (T_lim_)) is then calculated for each work bout and plotted against T_lim_. A simple linear regression analysis of the W_Lim_ versus T_Lim_ relationship gives a slope, defined as CP, and the y-intercept, previously described as an energy reserve, defined as the W′ (Figure 2a). Therefore, Moritani et al. [7] expanded on the previous work of Monod and Scherrer [6] in describing the linear relationship between time to exhaustion and work performed during whole-body cycle ergometry.

## 4. Critical Power Test Parameters

The physiological responses underlying the parameters of the CP test have been the subject of a number of studies over the last 40 years [7,8,9,10,14]. Based on the mathematical modeling, CP was described as “…the maximal power at which muscle can work without fatigue” [7] (p. 346). Theoretically, exercise performed above this power output can be maintained for a finite amount of time until exhaustion, while work below this power output was theorized to be able to be maintained indefinitely without fatigue (including both central and peripheral factors). In reality, no exercise can be completed without fatigue and, therefore, the duration of fatigueless work has been operationally defined as exercise that can be maintained for 30 to 60 min [8,16] under specific physiological conditions. More specifically, the CP is often defined as the highest power output that can be sustained for at least 30 min, where $\dot{V}$O_2_ and blood lactate reach steady state responses. This was supported through the demonstration that CP was correlated with the anaerobic threshold and was dependent on oxygen supply [7]. Additionally, it was demonstrated that $\dot{V}$O_2_ and blood lactate reached a delayed steady state for exercise at or below CP, but both physiological markers increased until exhaustion for exercise performed above CP [14]. Based on these responses, it has been suggested [16] that CP represents the demarcation, or separation, between the heavy and severe intensity domains. Generally, for untrained or recreationally trained individuals, this model overestimates the power output that meets this definition [17], but more closely approximates this power output for elite athletes due to differences in the presence of the $\dot{V}$O_2_ slow component phenomenon [16,18]. Thus, in actuality, CP is not a power output that can be maintained indefinitely without exhaustion but may reflect a power output that demarcates (with some error) differences in physiological responses and/or a transition phase between the heavy and severe intensity domains [19].

In addition to providing an individually derived performance threshold, the CP test provides a measure of an individual’s capacity to use stored energy supplies within the muscle (i.e., W′) as well as a method of predicting T_Lim_ for a power output above CP. The W′ reflects the total amount of work that can be performed above CP using only energy stored within the muscle (i.e., ATP bound to myosin, phosphocreatine, glycogen, and oxygen bound to myoglobin) before it is limited by exhaustion [7,9]. Based on the linear, 2-paramter regression equation from Moritani et al. [7] T_Lim_ = W′/(P − CP), where T_Lim_ equals time to exhaustion, W′ is the anaerobic work capacity, P is the imposed power output above CP, and CP is the derived critical power, a coach or practitioner can theoretically predict the time to exhaustion at a given power output above the CP due to intramuscular energy stores being used at a “predictable rate based on the magnitude of the difference between the imposed power loading (P) and CP” [17] (pp. 1001–1002).

## 5. Methodological Considerations

Although the linear, 2-parameter total work model is the most prevalent in the literature, there are five mathematical models that have been used to estimate CP [20]. These include 2- and 3-parameter models, as well as an exponential model, that demonstrate a hierarchical order of CP and W′ estimates (Figure 2). The exponential model typically produces the highest estimates of CP, followed by the linear and nonlinear, 2-parameter models, and the nonlinear, 3-parameter model typically produces the lowest estimates of CP. For the W′, the highest estimates are derived from the nonlinear, 3-parameter model and the lowest from the linear models [20,21,22,23]. The various estimates of CP from these models result in different physiological responses to continuous exercise at CP. Authors [21,23,24] have suggested that the linear, 2-parameter model may overestimate the true CP for untrained or recreationally trained subjects, however, the estimates of CP are more closely representative of a sustainable power output for highly trained endurance athletes [9,10,11]. This variance in the accuracy of the CP model may be due to inherent differences in the mathematical model used in the derivation of CP. Morton [21] suggested that the nonlinear, 3-parameter may more accurately reflect the true CP based on its ability to include the P_max_, maximal instantaneous power, which lowers the CP estimate [20,21]. Thus, the CP estimates from the linear, 2-parameter models may be more accurate for highly trained athletes, while the nonlinear, 3-parameter model may provide more accurate estimates for untrained or recreationally trained individuals. However, more research is needed on the nonlinear, 3-parameter model, which has been limited thus far by the complexity of the modeling and the challenges in elucidating the differences between physiology and mathematics.

Traditionally, the CP is determined from three to five exhaustive work bouts performed at various power outputs. However, the CP can be determined with only two work bouts but requires a skilled investigator and prior knowledge of an individual’s performance capabilities. It was suggested [25] that if only two work bouts are utilized, the time to exhaustion of the two rides should be separated by at least 5 min to minimize the standard error. In addition, there is some evidence that more highly trained individuals, such as collegiate endurance athletes, can accurately derive their CP from estimations of performance times for various distances [26,27]. Investigators have also explored the estimation of CP from a single, 3-min all-out test (3MT). This can be completed with [28,29] or without [30,31] a prior graded exercise test (GXT). The CP is estimated as the average end power (EP) over the final 30 s of the test, after the W′ has been depleted [28,29]. Therefore, the amount of work done above EP (WEP) is equal to the W′ [29]. Thus, CP can be estimated from multiple work bouts or from a single work bout, with both methods producing similar estimates of CP; however, the accuracy of the estimate may depend on the fitness level of the subject.

## 6. Progression of the Modeling across Exercise Modalities: Applications of the Critical Power Model to DCER Exercises

**Figure 3:**
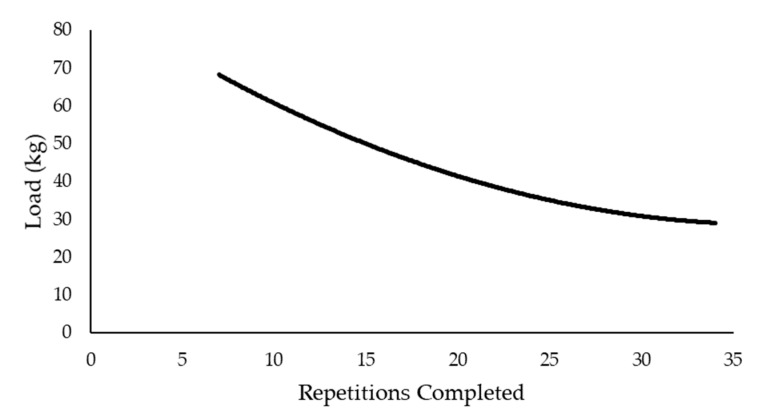
The hyperbolic relationship between load and total number of repetitions completed.

**Figure 4:**
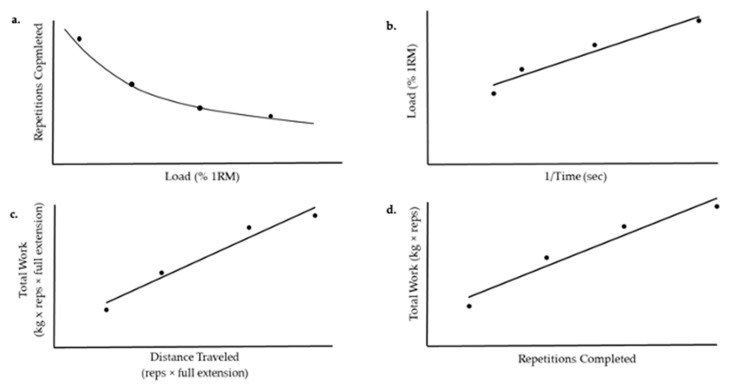
The mathematical models used to determine the critical load (CL) and L′ for dynamic constant external resistance exercise; (**a**) non-linear, 3-parameter regression model of repetitions completed versus load (% one repetition maximum [1RM]), where CL is the asymptote, L′ is the curvature constant, and maximal instantaneous lift (L_max_) is the x-intercept; (**b**) linear regression model of the load versus inverse of time, where L′ is the slope and CL is the y-intercept; (**c**) linear regression model of the total work versus distance traveled, where CL is the slope and L′ is the y-intercept; (**d**) linear regression model of the total work versus total repetitions completed, where CL is the slope and L′ is the y-intercept.

The original work of Monod and Scherrer [6] and Moritani et al. [7] led to the application of the CP concept to other modes of exercise, including running [13], swimming [32], and rowing [33], such that a linear relationship can be derived from the total work completed and time to exhaustion for each different modality. It is this body of work over the last 50 plus years that has expanded our understanding of the limits of human performance and led to the recent application of the CP model to DCER exercises, including the bench press [34], leg press [35], deadlift [36], and leg extension [37]. The same hyperbolic relationship that has been established across various continuous, whole-body, dynamic movements has also been demonstrated for upper-, lower-, and whole-body DCER exercises (Figure 3). For DCER exercise, the load is substituted for the power output or velocity and plotted against, time, distance, or number of repetitions completed. For DCER exercises, each repetition includes the concentric and eccentric phase of the lift. The asymptote of the load versus repetition (or duration) relationship is defined as the critical load (CL) and can be estimated from the same linear and non-linear mathematical models used to derive the CP [38] (Figure 4). At this time, there have been several terms used to define the asymptote of the load versus repetition relationship, including the critical lift [34], critical resistance [36,37], and critical load [35,38]. Because the term critical load most accurately reflects the DCER modality, and to be consistent with terminology across the literature, we recommend the use of the term critical load (CL) in future research.

The estimation of the CL is derived from the performance of repetitions to failure for three to four separate loads that are greater than the asymptote of the load versus repetition relationship. The CP model was first applied to the bench press [34]. The loads were selected so that task failure occurred within specified repetition ranges of 3–10 repetitions for the highest load, 10–20 and 21–40 repetitions for the middle two loads, and greater than or equal to 41 repetitions for the lowest load, which resulted in mean loads of ~85%, 69%, 51%, and 34% of the one repetition maximum (1RM), respectively [34]. The authors reported a nonlinear relationship between total work (load (kg) × number of repetitions) and the number of repetitions plotted using the linear, 2-parameter total work model. However, neither the goodness of fit (*r*^2^) values nor estimates of the CL were provided for the linear, 2-parameter total work model [34]. The nonlinearity appeared to be driven primarily by the total work performed at the lowest load (~34% 1RM, repetitions > 41) [24] (p. 156). Based on the observed nonlinear response for the linear, 2-parameter total work model, the authors [34] further examined the bench press performance with the nonlinear, 3-parameter model (Figure 4a). For this model, the asymptote of the repetition versus load relationship was defined as the CL. This modeling resulted in goodness of fit (*r*^2^) values that ranged from 0.6698–0.9999. Interestingly, the asymptote (i.e., CL) was reported to be 0 kg for most subjects (12 of 16) [34]. However, the load selections for the modeling in this study may have also contributed to these zero estimations for the CL from the nonlinear, 3-parameter model. Specifically, it is likely that the lowest load (~34% 1RM, repetitions > 41) was peri-asymptotic and likely lowered the estimates of the CL from this model. This initial application highlighted the importance of load selection in the estimation of the CL.

The application of the CP model to DCER exercise was further examined for the leg press [35]. For this movement, the total work was determined from the performance of repetitions to failure (and the time to failure was recorded) for load settings of 30%, 60%, 75%, and 90% of 1RM, and the linear, 2-parameter inverse time model was used to estimate the CL (Figure 4b). The modeling of the highest three loads resulted in a significantly greater estimate of the CL (53% 1RM) than the estimate of CL for all four loads (38% 1RM). In addition, the range of *r*^2^ values were higher for the three loads (range = 0.9512–0.9988) compared to the four loads (range = 0.7799–0.8909). The decreased linearity for the model utilizing all four loads may be explained by the use of a load (30% 1RM) below the estimated CL (38% 1RM). Like CP, the mathematical modeling of the CL relies on the assumption that loads are selected above the CL for its estimation. Peri-asymptotic loads will decrease the linearity and accuracy of the estimation of the CL. Although loads between 30% and 80% maximal voluntary contraction (MVC) force may be appropriate for isometric exercise and the determination of critical torque or critical force [6,39], 30% of 1RM appears to be too low in these initial DCER applications, as evidenced by CL estimates that range from ~25% and 40% 1RM [35,36,37]. This may be related to the intermittent nature of DCER exercise that allows for some restoration of blood flow during the eccentric phase and/or between repetitions (depending on the cadence).

To address the nonlinearity around the lowest load setting identified in these initial applications of the CP model to DCER exercise, Dinyer et al. [36] used loads set at 50%, 60%, 70%, and 80% 1RM for the deadlift and the leg extension (Figure 4c,d). The *r*^2^ values ranged from 0.864–0.989. Of particular importance was the fact that the lowest load was above the asymptote, which corresponded to 40% 1RM for the deadlift [36] and 26% 1RM for the leg extension [37]. The high linearity between total work (load (kg) × repetitions) and repetitions completed for both DCER exercises highlighted the necessity for choosing loads that are above the asymptote for the derivation of the CL. Thus, there are methodological considerations for the mathematical modeling of DCER exercise that have been identified in these initial applications [34,35,36,37,38] that underlie the importance of selecting loads that are neither too high nor too low (peri-asymptotic), which can result in the loss of linearity in the total work versus repetition relationship and decrease the validity of the CL estimation.

## 7. Additional Methodological Considerations for the Determination of the CL Test Parameters

In addition to the load selection, the cadence of the repetitions to failure is an important consideration in the modeling of DCER exercise. At this time, investigators [34,35,36,37,38] have controlled the cadence in the initial applications of the model. The specific cadence has varied across exercises, from 1.1 to 1.5 s per contraction phase (i.e., concentric and eccentric), depending on the specific nature of the movement. The selection of a cadence was specified based on pilot testing to determine a rate that allowed for smooth, continuous repetitions through both the concentric and eccentric phases. Currently, however, there is limited evidence on variability in cadence selection or self-selected pacing strategies for DCER exercises and the subsequent effects on the CL estimates.

The effects of lifting method, and indirectly the cadence, on the CL estimates were demonstrated in a recent methodological study of the CL test for the deadlift [40]. Specifically, the touch-and-go (TG) versus reset (RS) method were compared in the estimations of the CL for four separate loads (50–80% 1RM). The cadence was controlled for the concentric and eccentric phase (1.33 s for each phase) of both methods. However, the RS method was distinguished from the TG with the addition of a 1.33 s pause between each repetition. The mean CL estimates were not different between the two methods (TG = 38% 1RM and RS = 37% 1RM), however, there was a wide range in CL estimates for individual subjects. That is, most subjects performed decidedly better in one method compared to other, and the individual differences between the CL from the RS method versus the CL from the TG method ranged from −8.8 kg to 17.0 kg [40]. It was hypothesized [40] that these individual differences may reflect muscle group-specific fatigue responses, where the TG method was reported to affect the muscles of the forearm and grip on the bar, while the RS method resulted in more pronounced low-back fatigue. These muscle specific fatigue responses were anecdotal reports but suggested an important area for future research. Specifically, future studies should examine these muscle group-specific fatigue responses on the estimation of the CL and the subsequent performance of repetitions at the CL to provide the best method for determination that is based on the specific nature of each exercise.

## 8. Test Parameters: Critical Load and the y-Intercept (L′)

Based on the mathematical model, theoretically, the CL reflects highest load that can be lifted indefinitely or “… sustained for a very long time without fatigue…” [6] (p. 329). However, just as the CP does not truly reflect a fatigueless power output, the CL is not truly a load that can be lifted indefinitely. The CL has been operationally defined as the highest load that can be lifted for at least 30 to 50 repetitions, depending on the muscle action [35,36] and demarcates physiological responses for DCER exercise performed to failure [37].

In the first application to DCER exercise, the CL was defined as the asymptote of the load versus duration relationship from the nonlinear, 3-parameter model [34]. In this study, however, the CL was estimated to be 0 kg for 12 of the 16 subjects [34]. It was suggested that these zero estimations reflect the anaerobic nature of DCER exercise and negligible contributions of aerobic metabolism. This hypothesis, however, assumes the direct analogy to continuous, whole-body dynamic exercise like cycling and running. Specifically, for cycle ergometry exercise, Moritani et al. [7] demonstrated that the CP was sensitive to hypoxia, but the W′ (y-intercept) remained unchanged. These findings informed the hypothesis that CP reflected the highest power output that could be maintained with reliance on aerobic energy reconstitution, while the W′ reflected the work performed using stored energy sources within the working muscle that were independent of oxygen supply (i.e., blood flow). These metabolic and circulatory system interpretations of CP and W′ for whole-body, continuous dynamic exercise, however, cannot be directly applied to DCER exercise because aerobic metabolism does not contribute to DCER exercise to the same degree as during cycle ergometry or running. Thus, it is unlikely the CL reflects a load that can be performed relying only on aerobic energy production.

**Figure 5:**
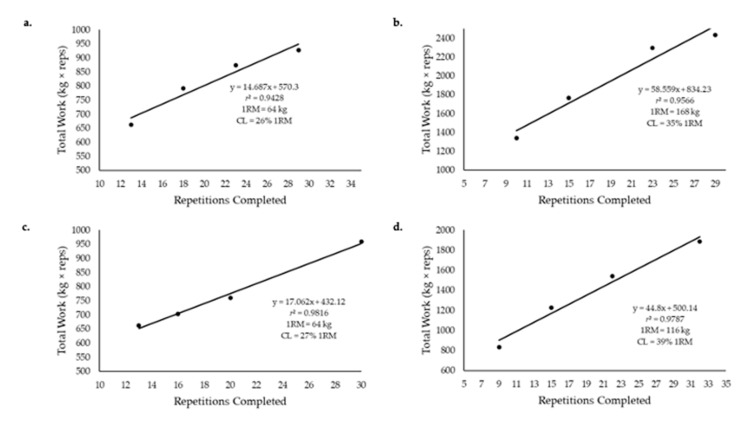
A representative example of the mathematical model used to derive the critical load (CL) and L′ for a male and female subject for deadlift and leg extension exercises. The CL is slope and L′ is the y-intercept; (**a**) leg extension responses for a male subject; (**b**) deadlift responses for a male subject; (**c**) leg extension responses for a female subject; (**d**) deadlift responses for a female subject.

At this time, the physiology underlying performance above and below the CL is not well understood, although there is some evidence of unique neuromuscular responses [37] above and below the CL. Specifically, performance of repetitions to failure above the CL resulted in an earlier increase in muscle activation compared to performance below the threshold, while decreases in motor unit action potential conduction velocity (MUAP CV) occurred at 90% of the total repetitions to failure above the CL, but 50% of repetitions to failure below the CL [37]. The authors [37] hypothesized that this may be due greater increases in muscle activation for continued force development that leads to increases in metabolite accumulation, which decreases the MUAP CV signal, and compromises in local blood flow when repetitions are performed above the CL. Conversely, when repetitions were performed below the CL, increases in muscle activation occurred near the end of the repetitions to failure and decreases in MUAP CV occurred midway through the performance of repetitions to failure, which was likely due to a better ability to withstand metabolite accumulation stemming from better blood flow when performed at a lower load. While this indicates that the CL demarcates a fatigue threshold where neuromuscular responses differ when repetitions are performed above and below the CL, the specific physiological phenomena dictating these responses has not been fully examined. Interestingly, in their original work, Monod and Scherrer [6] demonstrated distinct threshold responses for continuous isometric versus intermittent isometric exercise for local muscle actions (less than one-third of the total muscle mass). The asymptote of load-duration relationship for continuous isometric exercise was lower (14% MVC force) than that for intermittent isometric exercise (40% MVC) [6]. This difference was hypothesized to be related to the circulatory conditions within the muscle, where continuous isometric exercise does not allow for restoration of blood flow between contractions as does intermittent exercise. This blood flow alteration would likely happen at the level of the capillary, which would limit removal of metabolic byproducts as well as the delivery of oxygen to the muscle. This blood flow alternation may explain the asymptotic nature of the load versus duration relationship for DCER exercise performed to failure. In support of this hypothesis, the % 1RM of the CL has been shown to be greater for the whole-body deadlift exercise (~40% 1RM), where a pause between the concentric and eccentric phases resulted in an exercise more consistent with intermittent isometric exercise compared to local, bilateral leg extension exercise (~26% 1RM) without a defined pause between repetitions [36,37] (Figure 5). Furthermore, women have been shown to have a higher relative CL compared to men for whole-body DCER exercises (Figure 5b,d), but not local muscle actions (Figure 5a,c), which may be related to differences in muscle size and, thus, intramuscular pressures that alter blood flow [41]. Thus, it appears the CL is specific to the muscle action (muscle group) and cadence for DCER exercise, and the modeling may also be sensitive to detect sex differences in submaximal performance capabilities.

There are no studies that have examined the potential physiological or performance implications of the y-intercept. This parameter has previously been described as the anaerobic lift capacity [34]. However, based on the current lack of physiological understanding of this parameter, we recommend the use of the term lift’ (L′). This terminology has been used to describe the y-intercept as the curvature constant of the load versus duration relationship. If the CL defines the highest load that can be performed before blood flow is compromised (altered) within the working muscle, it is possible that the L′ reflects the total amount of work that can be performed above the CL, without blood flow within the muscle (capillary occlusion). Future studies should investigate the metabolic and circulatory responses during performance above and below the CL to distinguish the physiological mechanisms underlying the L′ parameter.

## 9. Research and Training Applications of the Critical Load Model

The question of the resistance training load necessary for skeletal muscle adaptation (strength/hypertrophy/performance) has been of increasing interest [42,43,44,45,46]. It has been generally held that higher loads (>70% 1RM) are required to maximize strength and hypertrophy adaptations [47]. However, recent comparisons of lower (30–50%) and higher (70–90%) load training to failure have yielded mixed results [42,43,44,45,46]. Specifically, authors have reported equivalent increases in maximal strength in trained men and untrained men and women for the back squat, bench press, and machine weight exercises [42,45,46], while inferior increases in maximal strength were reported for lower loads compared to higher loads during isolated, single-group muscle actions such as the forearm flexion and leg extension [43,44]. Furthermore, while time under tension and total volume accumulation have been reported as greater when training to failure at lower loads [42,43,45,46], muscle activation does not reach maximal levels when repetitions are completed to failure at lower loads, compared to higher loads [48]. This variability in strength adaptations may be, in part, related to where the lower load is performed relative to the CL. The CL has been shown to vary from ~26% 1RM to 50% 1RM, depending on the muscle action [35,36,37]. Thus, it is likely that, for at least some of the subjects in a sample, the low load training may have been performed below the CL and contributed to the variability in responses observed at lower training loads. Although the precise physiology underlying performance above and below this threshold is still unknown, the asymptotic nature of the load versus repetitions relationship indicates distinct responses above and below the CL. It is possible that training below the CL results in submaximal levels of muscle activation, so that not all muscle fibers are subjected to the training stimulus, and thus individuals training below their CL are not able to maximize strength and/or hypertrophic adaptations. While there is no evidence to support training at or above the CL for strength and hypertrophic adaptations, the use of this modeling may provide a method to examine fatigue that is based on fatigue characteristics specific to each individual’s capabilities. Therefore, this model may provide an estimate of the lowest load that can be used for each individual to maximize strength adaptations for DCER exercises.

## 10. Recommendations for the Determination of the Critical Load

The following represent the current recommendations for the determination of the CL and L′, based on the available works on this modeling [34,35,36,37,38].

1. At least four loads are recommended for the determination of the CL and L′, and each load used in the mathematical model should be greater than the CL. At this time, 50% 1RM or greater is recommended for the lowest load, and under most conditions, increases in loads should be made at increments of 10% (i.e., 50%, 60%, 70%, 80% 1RM).
2. A cadence should be selected specific to each movement and standardized across subjects. This cadence should allow for successful completion of repetitions through the full range of motion for the lowest and highest loads.
3. For subjects unfamiliar with performing repetitions to failure, a familiarization session at a submaximal load around 50–60% may improve the accuracy of the modeling.
4. The model should be examined for each subject, and the *r*^2^ of the total work versus repetition relationship should be at least 0.75 or greater.
5. If an *r*^2^ is lower than 0.75 or the lowest load used in the model is lower than the CL for an individual subject, that load should be eliminated and an additional load setting greater than 50% 1RM should be performed and used in the analyses.
6. The CL and L′ can be estimated using the linear, 2-parameter total work (load (kg) × repetitions) versus duration relationship, the linear, 2-parameter load versus the inverse duration, or the nonlinear, 3-parameter model, and the duration should be expressed as repetitions.
7. The mean and range of *r*^2^ and standard error of the estimate (*SEE*) values from the regression model should be reported in all future works.

## 11. Future Research on the Critical Load Model

The following are important methodological and mechanistic questions to examine in future research. This is not an exhaustive list, but merely a basis from which to work.

1. Load selections—A wider range (e.g., 35–40% 1RM to 95% 1RM) of relative load settings should be examined across whole-body, upper-body, and lower-body, unilateral and bilateral muscle actions to determine the effects of the load setting on the mathematical modeling.
2. Number of loads—The effects of using two loads versus three, four, or five loads on the parameter estimates CL and L′ from the linear and non-linear mathematical models should be examined.
3. Effects of cadence—The effect of various cadences, including a self-selected cadence, on the estimation of the CL and L′ should be examined.
4. Reliability—Future studies should examine the reliability of the CL and L′ for various DCER exercises.
5. Muscle specific thresholds—The CL model should be examined for agonist versus antagonist muscle actions, bilateral versus unilateral muscle actions, and upper- versus lower-body muscle groups to determine if the mathematical model is sensitive to detect muscle group-specific fatigue characteristics.
6. Mode-specific thresholds—Studies should compare the parameter estimates for isometric versus DCER movements in the same muscle group.
7. Physiological underpinnings—Further investigation is warranted to examine the potential metabolic and circulatory factors underlying the determination of the CL and L′ as well as the prediction of performance using the CL model.
8. Training studies—Training adaptations for strength and hypertrophy should be examined for loads prescribed above and below the CL for each individual.

## Data Availability

No new data were created or analyzed in this study. Data sharing is not applicable to this article.
